# Machine Learning–Based Prediction of Attention-Deficit/Hyperactivity Disorder and Sleep Problems With Wearable Data in Children

**DOI:** 10.1001/jamanetworkopen.2023.3502

**Published:** 2023-03-17

**Authors:** Won-Pyo Kim, Hyun-Jin Kim, Seung Pil Pack, Jae-Hyun Lim, Chul-Hyun Cho, Heon-Jeong Lee

**Affiliations:** 1 LumanLab Inc, R&D Center, Seoul, South Korea; 2 Department of Psychiatry, Chungnam National University Sejong Hospital, Sejong, South Korea; 3 Department of Biotechnology and Bioinformatics, Korea University, Sejong, South Korea; 4 Department of Psychiatry, Korea University College of Medicine, Seoul, South Korea; 5 Department of Biomedical Informatics, Korea University College of Medicine, Seoul, South Korea; 6 Chronobiology Institute, Korea University, Seoul, South Korea

## Abstract

**Importance:**

Early detection of attention-deficit/hyperactivity disorder (ADHD) and sleep problems is paramount for children’s mental health. Interview-based diagnostic approaches have drawbacks, necessitating the development of an evaluation method that uses digital phenotypes in daily life.

**Objective:**

To evaluate the predictive performance of machine learning (ML) models by setting the data obtained from personal digital devices comprising training features (ie, wearable data) and diagnostic results of ADHD and sleep problems by the Kiddie Schedule for Affective Disorders and Schizophrenia Present and Lifetime Version for *Diagnostic and Statistical Manual of Mental Disorders, 5th edition* (K-SADS) as a prediction class from the Adolescent Brain Cognitive Development (ABCD) study.

**Design, Setting, and Participants:**

In this diagnostic study, wearable data and K-SADS data were collected at 21 sites in the US in the ABCD study (release 3.0, November 2, 2020, analyzed October 11, 2021). Screening data from 6571 patients and 21 days of wearable data from 5725 patients collected at the 2-year follow-up were used, and circadian rhythm–based features were generated for each participant. A total of 12 348 wearable data for ADHD and 39 160 for sleep problems were merged for developing ML models.

**Main Outcomes and Measures:**

The average performance of the ML models was measured using an area under the receiver operating characteristics curve (AUC), sensitivity, specificity, positive predictive value (PPV), and negative predictive value (NPV). In addition, the Shapley Additive Explanations value was used to calculate the importance of features.

**Results:**

The final population consisted of 79 children with ADHD problems (mean [SD] age, 144.5 [8.1] months; 55 [69.6%] males) vs 1011 controls and 68 with sleep problems (mean [SD] age, 143.5 [7.5] months; 38 [55.9%] males) vs 3346 controls. The ML models showed reasonable predictive performance for ADHD (AUC, 0.798; sensitivity, 0.756; specificity, 0.716; PPV, 0.159; and NPV, 0.976) and sleep problems (AUC, 0.737; sensitivity, 0.743; specificity, 0.632; PPV, 0.036; and NPV, 0.992).

**Conclusions and Relevance:**

In this diagnostic study, an ML method for early detection or screening using digital phenotypes in children’s daily lives was developed. The results support facilitating early detection in children; however, additional follow-up studies can improve its performance.

## Key Points

**Question:**

Can data obtained from personal digital devices (ie, wearable devices) in children collected from the Adolescent Brain Cognitive Development study be used for predicting attention-deficit/hyperactivity disorder (ADHD) and sleep problems?

**Findings:**

In this diagnostic study of 79 children with ADHD and 68 children with sleep problems, circadian rhythm–based wearable data were useful for developing a suitable machine learning model. The model showed reasonable predictive performance.

**Meaning:**

The findings of this diagnostic study suggest that wearable data have the potential to detect ADHD and sleep problems; this approach can facilitate the application of digital phenotypes in daily life for mental problems in children.

## Teaser

This diagnostic study develops a machine learning model using data from wearable digital devices to detect attention-deficit/hyperactivity disorder (ADHD) and sleep problems in children.

## Introduction

Mental health problems are prevalent in childhood and are major health and social burdens. The prevalence of mental problems in children and adolescents worldwide is approximately 10% to 20%, with most symptoms appearing before age 14 years.^1,2^ Psychiatric disorders diagnosed in childhood have a high correlation with lifelong mental problems, making early detection and therapeutic intervention vital.^2,3,4^

Attention-deficit/hyperactivity disorder (ADHD) is prevalent in approximately 5% of children, and sleep problems are prevalent in approximately 10% of children.^3,5,6^ Both ADHD and sleep problems have multifactorial etiologic factors that can cause neurocognitive and functional impairment and can have negative effects in several areas, such as educational achievements, socioeconomic outcomes, and parent-child relationships.^7,8^ Currently, the diagnosis of ADHD and sleep problems is not based on measurable methods, such as biomarkers, but is diagnosed through clinician evaluation by confirming the severity, frequency, and duration of symptoms.^2^ Structured interview tools and semistructured interview tools are recommended for assessing psychiatric disorders in children.^9,10^ These evaluation tools, used by trained professionals, have limitations during face-to-face evaluations due to time and spatial constraints, as well as differences in results among the interviewees.^11^

The development of personal digital devices has enabled moment-by-moment collection of individual-level behavioral and physiologic data and their integration and analysis for health care as digital phenotypes.^12^ Considering the advantage of enabling digital phenotypes in diagnoses and treatments for personalized health services, the digital health care market is expected to grow exponentially.^13^ In particular, digital phenotypes from personal digital devices can provide insights into an individual’s real-time mental state.^14,15^ Although recent studies have reported their advantages, few studies have used digital phenotypes for psychiatric diagnosis.^16^ Conventional assessment methods of mental states through questionnaires or interviews vary regarding data quality and have limitations such as recall bias. Therefore, there is a demand for a new diagnostic method based on quantitative data by using real-time digital phenotypes gathered from people’s daily lives.^17^

The Adolescent Brain Cognitive Development (ABCD) study is a longitudinal observational study of more than 10 000 adolescents recruited from 21 locations across the US.^18^ It aims to trace the trajectory of brain development in children and adolescents and investigate the factors influencing the onset, course, and severity of various psychiatric disorders.^19,20,21^ Several studies have used data from the ABCD study to investigate mental disorders in children and adolescents.^20,21,22^ Particularly, the ABCD study provides wearable data (Fitbit Wearable Wrist Tracker, Google LLC) on children’s physical activity, sleep, and heart rates. Accordingly, the present study aimed to develop a machine learning (ML) method to predict ADHD and sleep problems in children using wearable data, anthropometrics, and the Kiddie Schedule for Affective Disorders and Schizophrenia Present and Lifetime Version for *Diagnostic and Statistical Manual of Mental Disorders, 5th edition* (K-SADS) data from the ABCD study.

## Methods

### Study Design and Setting

The ABCD study is a large-scale, longitudinal study involving children aged 9 to 11 years from 21 data collection sites,^23^ covering 17 states in the US. It was designed considering the demographic composition of the US and using a school-based recruitment strategy based on epidemiologic information.^18^ Starting with the initial 11 878 patients, data for 11 235 patients have been released at 1-year and for 6571 patients at 2-year follow-ups. The ABCD study data are available at the National Institute of Mental Health data repository. This study used screening data from 6571 patients and 21 days of wearable data (ABCD mobile tech) from 5725 patients released at the 2-year follow-up. The study was reviewed and approved, with a waiver of informed consent, by the institutional review board of Chungnam National University Sejong Hospital and followed the Transparent Reporting of a Multivariable Prediction Model for Individual Prognosis or Diagnosis (TRIPOD) reporting guideline.

### Participants

The study participants were selected using a diagnosis label on the K-SADS from parents and children without comorbidities. The exclusion criteria were reported comorbidities and missing or abnormal values in wearable data. Detailed inclusion and exclusion criteria are described in eMethods 1 in Supplement 1. Following the K-SADS parent data, the ADHD diagnoses showed various symptoms, such as explosive irritability, avoiding tasks requiring attention, difficulty sustaining attention since elementary school, easily distracted, and interference with social, academic, or occupational functioning. Following the K-SADS child data, the sleep problems diagnosis only showed insomnia symptoms.

### Procedures

#### Data Collection and Feature Extraction

This study used data regarding participants’ sex, wearable device, anthropometrics of youth, and K-SADS data from the ABCD study (release 3.0, November 2, 2020, analyzed October 11, 2021) provided by the National Institute of Mental Health database.^10^ The data were expanded to reflect participants’ daily circadian rhythms through cosinor analysis^24^ using physical biomarkers (age, height, and weight in anthropometrics) and wearable data including 9 variables (heart rate, stage of sleep for both the 30- and 60-second records, calories, intensity, and metabolic equivalents in the ABCD mobile tech). The list of generated features is given in Table 1. The features were generated from circadian rhythm–based physical biomarkers, including arithmetic variables, calorie consumption, a difference of basal metabolic rates, cosinor analysis-related variables, the least active 5-hour periods, and the most active 10-hour periods.^25,26^ To generate valid features, the wearable data recorded for less than 30 minutes per hour and less than 5 hours per day were regarded as invalid data in this study. In addition, a total of 64 circadian features were created, including bedtime and daytime heart rates and step counts, duration of naps, and duration of sleep. eMethods 2 and equations 1 to 6 in the eAppendix in Supplement 1 present the detailed process and formula of generating the features.

**Table 1.** Characteristics of the Training Data Set for Attention-Deficit/Hyperactivity Disorder and Sleep Problems

Variables	Mean (SD)					
	Data set for ADHD			Data set for sleep problems		
	Control (n = 1011)	ADHD (n = 79)	*P* value	Control (n = 3346)	Sleep problems (n = 68)	*P* Value
**Demographic characteristic**						
Sex, No. (%)						
Male	513 (50.7)	55 (69.6)	<.001	1725 (51.6)	38 (55.9)	.007
Female	498 (49.3)	24 (30.4)		1621 (48.4)	30 (44.1)	
**Daily summarized features based on the wearable data**						
30-s–related features, min						
Duration						
Of being asleep	217.1 (36.3)	213.3 (37.2)	.003	217.9 (36.9)	217.0 (36.0)	.60
Of in-bed sleep	245.7 (41.3)	241.8 (42.2)	.007	246.4 (41.7)	247.4 (40.7)	.39
Of deep sleep	41.7 (17.3)	40.7 (17.7)	.14	41.6 (17.7)	41.7 (16.6)	.77
Of light sleep	127.1 (27.0)	123.8 (26.2)	.001	127.6 (26.9)	128.9 (27.2)	.20
Of REM sleep	48.3 (15.2)	48.8 (16.0)	.58	48.7 (15.3)	46.5 (15.4)	<.001
Of waking for short periods	28.6 (9.8)	28.5 (9.9)	.80	28.5 (9.7)	30.5 (9.5)	<.001
Of being asleep during a nap	37.5 (37.2)	39.0 (38.2)	.25	37.1 (36.5)	47.0 (40.7)	<.001
Of being in bed during a nap	44.9 (42.6)	46.4 (44.0)	.42	44.7 (41.9)	55.9 (46.5)	<.001
Rate, %						
Of deep sleep	19.0 (7.0)	18.8 (7.2)	.50	18.9 (7.1)	19.1 (6.9)	.48
Of light sleep	58.6 (8.4)	58.2 (8.5)	.19	58.7 (8.5)	59.4 (8.1)	.01
Of REM sleep	22.3 (6.5)	22.9 (6.7)	.02	22.4 (6.4)	21.5 (6.6)	<.001
Of wake	13.3 (4.3)	13.4 (4.4)	.28	13.2 (4.2)	14.1 (4.2)	<.001
Duration						
Of deep sleep stage	83.4 (34.7)	81.5 (35.5)	.14	83.3 (35.4)	83.3 (33.2)	.77
Of light sleep stage	254.2 (54.1)	247.5 (52.5)	.001	255.2 (53.9)	257.7 (54.3)	.20
Of REM sleep stage	57.3 (19.7)	57.0 (19.7)	.80	97.3 (30.6)	92.9 (30.9)	<.001
Of being wake stage	96.6 (30.5)	97.6 (32.0)	.58	57.0 (19.3)	60.9 (18.9)	<.001
Quality of sleep, %	86.7 (4.3)	86.6 (4.4)	.04	86.8 (4.2)	85.9 (4.2)	<.001
60-s–related features, min						
Duration						
Of being asleep	473.9 (76.4)	465.4 (79.8)	.001	475.6 (77.0)	477.1 (73.4)	.47
Of restlessness	28.4 (15.1)	28.8 (15.0)	.36	27.7 (15.0)	29.8 (14.8)	<.001
Of being wake	2.6 (4.6)	2.8 (3.8)	.06	2.6 (4.9)	2.6 (3.8)	.30
Quality of sleep, %	93.9 (3.1)	93.7 (3.1)	.04	94.0 (3.1)	93.6 (3.0)	<.001
Calorie-related features, kcal						
Difference						
Between Harris-Benedict and BMR^a^	−699.4 (411.6)	−769.2 (450.6)	<.001	−680.2 (506.5)	−661.7 (379.5)	.18
Between Katch-McArdle and BMR^a^	−842.0 (416.4)	−902.1 (457.6)	<.001	−826.8 (446.0)	−804.6 (381.3)	.13
Between Mifflin-St Jeor and BMR^a^	−352.5 (409.0)	−404.3 (449.6)	<.001	−337.4 (449.3)	−314.7 (373.8)	.09
Sum of calories	2050.9 (489.3)	2120.3 (517.4)	<.001	2023.9 (487.4)	2026.2 (437.5)	.35
Heart rate–related features, bpm						
Acrophase^b^	11.1 (16.4)	11.5 (15.4)	.26	11.2 (16.6)	11.4 (12.8)	.80
Amplitude^b^	12.0 (4.2)	12.4 (4.3)	.83	12.1 (4.2)	12.4 (4.3)	.17
MESOR^b^	82.7 (7.5)	84.4 (8.0)	<.001	83.3 (7.5)	83.7 (8.2)	.20
Goodness of fit, %^b^	45.7 (19.0)	46.9 (19.1)	.04	46.3 (19.1)	48.3 (19.0)	.002
Bedtime						
Maximum	110.1 (15.0)	111.3 (15.7)	.07	110.4 (15.0)	112.6 (14.7)	<.001
Minimum	58.3 (6.8)	58.9 (7.0)	.23	58.7 (6.8)	58.9 (6.9)	.36
Mean	72.8 (8.5)	74.4 (9.0)	<.001	73.2 (8.5)	74.1 (9.1)	.04
Daytime						
Maximum	137.5 (16.3)	139.2 (16.6)	.001	137.4 (15.9)	137.6 (15.5)	.51
Minimum	59.8 (6.3)	59.8 (6.4)	.12	60.0 (6.3)	59.8 (6.5)	.30
Mean	88.4 (8.7)	89.9 (9.4)	<.001	89.0 (8.7)	89.0 (9.3)	.94
Maximum	101.8 (7.6)	103.5 (8.5)	<.001	102.2 (7.6)	102.9 (8.0)	.11
Minimum	69.1 (7.3)	70.2 (7.7)	.003	69.6 (7.4)	70.0 (7.9)	.18
Mean	82.4 (8.0)	84.0 (8.5)	<.001	83.0 (8.0)	83.4 (8.4)	.27
Variance	240.7 (105.7)	247.1 (111.0)	.10	241.3 (103.5)	242.2 (99.3)	.70
Standard deviation	15.2 (3.3)	15.3 (3.5)	.10	15.2 (3.2)	15.3 (3.1)	.70
Intensity-related features						
Maximum	2.3 (0.9)	2.3 (0.9)	.05	2.2 (0.9)	2.1 (0.9)	.01
Minimum^c^	0.0 (0.0)	0.0 (0.0)	>.99	0.0 (0.0)	0.0 (0.0)	>.99
Mean	0.3 (0.1)	0.3 (0.1)	.03	0.3 (0.1)	0.2 (0.1)	.007
MET-related features, min						
Maximum	7.1 (1.6)	7.0 (1.4)	.28	7.1 (1.6)	7.1 (1.5)	.43
Minimum	1.0 (0.0)	1.0 (0.0)	.42	1.0 (0.0)	1.0 (0.0)	.31
Average	1.6 (0.3)	1.6 (0.3)	<.001	1.6 (0.3)	1.6 (0.3)	.03
Duration						
Of light activity	105.6 (39.1)	99.1 (38.7)	<.001	106.4 (39.9)	108.7 (38.7)	.20
Of moderate activity	196.6 (92.0)	211.7 (104.4)	<.001	198.3 (91.3)	194.2 (87.3)	.23
Of sedentary activity	385.7 (108.8)	393.8 (119.4)	.17	384.4 (111.2)	405.9 (103.3)	<.001
Of vigorous activity	15.0 (23.0)	17.8 (25.1)	.007	14.6 (22.7)	11.5 (20.1)	<.001
Steps-related features, steps						
Acrophase^b^	112.7 (27.3)	114.5 (27.1)	.009	113.0 (27.4)	113.8 (33.7)	.40
Amplitude^b^	444.7 (202.8)	475.4 (228.9)	.53	442.7 (197.3)	408.2 (206.2)	.10
MESOR^b^	397.4 (166.0)	412.2 (185.3)	.08	395.1 (160.3)	364.1 (162.9)	<.001
Goodness of fit, %^b^	29.2 (10.9)	30.0 (10.9)	.03	29.6 (10.9)	30.3 (10.4)	.04
Sum of bedtime	543.0 (737.7)	541.2 (749.1)	.26	545.8 (752.5)	564.5 (612.1)	<.001
Sum of daytime	9111.4 (4952.7)	9585.7 (5620.1)	.09	9039.5 (4824.0)	8221.0 (4770.3)	<.001
Sum of steps^d^	9654.3 (5041.8)	10127.0 (5750.4)	.11	9585.3 (4918.1)	8785.5 (4811.5)	<.001
IS, 0-2	0.2 (0.2)	0.2 (0.3)	<.001	0.2 (0.2)	0.2 (0.2)	.03
IV, 0-1	1.0 (0.4)	1.0 (0.4)	.003	1.0 (0.4)	1.0 (0.4)	.92
L5, h	1.7 (3.9)	1.7 (3.5)	.18	1.7 (4.1)	2.1 (4.0)	<.001
M10	846.8 (412.5)	911.9 (463.0)	<.001	840.4 (402.5)	770.6 (409.6)	<.001
RA, 0-1	1.0 (0.0)	1.0 (0.0)	.33	1.0 (0.0)	1.0 (0.0)	<.001

Abbreviations: ADHD, attention-deficit/hyperactivity disorder; BMR, basal metabolic rate; IS, interdaily stability; IV, intradaily variability; L5, least-active 5-hour period; M10, most active 10-hour period; MESOR, midline estimating statistics of rhythm; MET, metabolic equivalent; RA, relative activity; REM, rapid eye movement.

^a^ Detailed equations are listed in eEquations 1 to 3 in the eAppendix in Supplement 1. Negative values for these features indicate that the daily calorie consumption is higher than the estimated BMR.

^b^ These are the estimated values of the cosinor analysis to reflect the circadian rhythm. Goodness of fit represents how much it fits the cosine graph of the 24-hour period in %.

^c^ The intensity daily minimum was recorded as 0.0 during sleep, making it impossible to calculate the *P* value.

^d^ The equation for each value is shown in the eAppendix in Supplement 1.

#### Training Data Set

**Figure 1.**
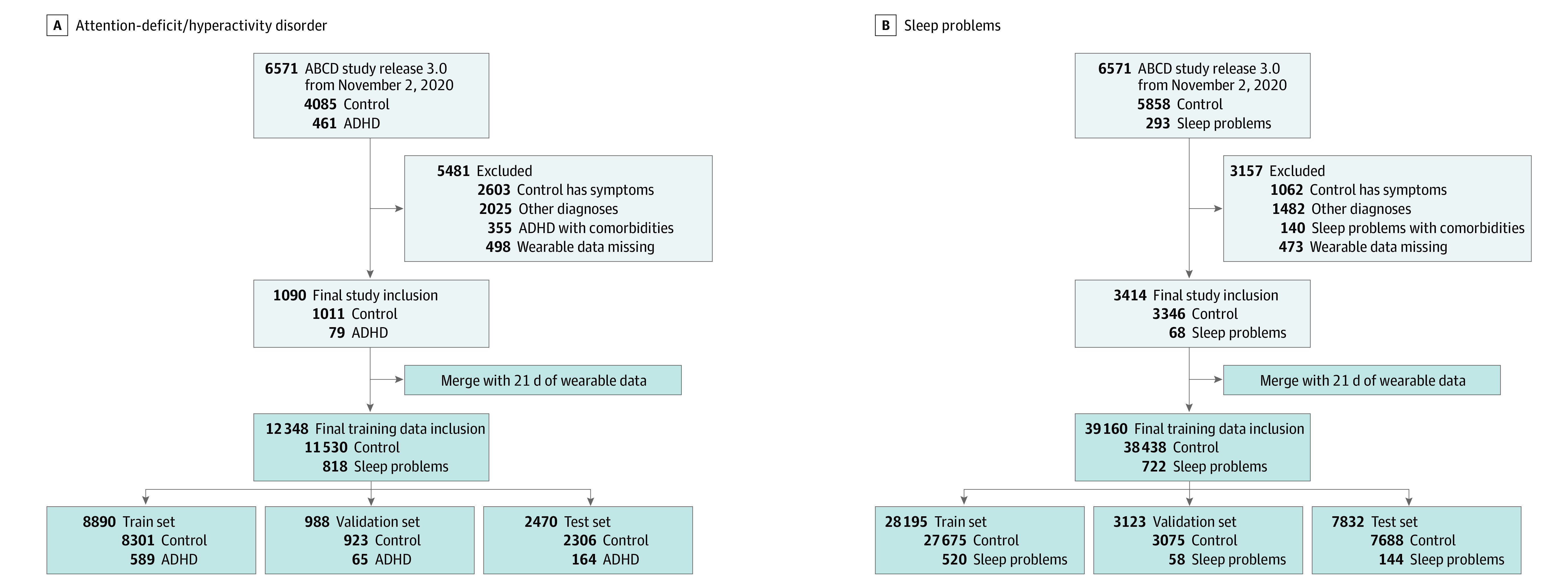
Study Population and Formation of Training Dataset Attention-deficit/hyperactivity disorder (A) and sleep problems (B) populations. The final training data inclusion represents the generated wearable data linked (merged) with each corresponding diagnosis in individuals following a unique identifier. ABCD indicates Adolescent Brain Cognitive Development; ADHD, attention-deficit/hyperactivity disorder.

The wearable data (mobile tech in the ABCD study) comprised 5275 individuals. Data on the children selected for analysis were included. These individuals were used for linking 21 days of the wearable data using a unique identifier (subjectkey in the ABCD study) to generate the training data set. Figure 1 shows the study population and the amount of the training data for each group, and eMethods 3 in Supplement 1 describes the detailed procedure for making the training data sets.

### Statistical Analysis

Python software, version 3.7.10 (Python Software Foundation), was used for all analyses. Categorical variables are presented as count (percentage); continuous variables are presented as mean (SD). After conducting the Kolmogorov-Smirnov test to determine the distribution of the data set, the independent sample test for gaussian or the Mann-Whitney test for nongaussian distribution was used. Statistical significance was indicated by a 2-sided *P* value <.05 in this study.

The training data set was split into 70% for the training set, 10% for the validation set, and 20% for the hold-out test set. Considering that the proportion of the prediction class was highly imbalanced at 6.62% for ADHD and 1.84% for sleep problems in the training data set, the training set comprised a strategy of the hyperensemble Smote Undersampled Random Forest (hyperSMURF) algorithm.^27^ The hyperSMURF generates the training set by dividing the imbalanced data into N partitions with a maintained ratio (eg, a stratified k-fold) and balancing the ratio using the synthetic minority oversampling technique for the minority class and undersampling for the majority class. For predicting each diagnosis, the ML models consisted of random forest, extreme gradient boosting, and light gradient-boosting machine (LightGBM) that were generated 50 times to estimate an average performance. The model evaluation metrics included area under the receiver operating characteristic curve (AUC), sensitivity, specificity, positive predictive value (PPV), and negative predictive value (NPV). The best model was estimated using the validation set and measured PPV. The Shapley Additive Explanations values were calculated to determine the importance of features.^28,29,30^

## Results

### Participants

Of the 6571 children with screening data, a total of 1090 individuals (16.6%) were included for the ADHD analysis, 79 (1.2%) as diagnoses (mean [SD] age, 144.5 [8.1] months; 55 [69.6%] males) and 1011 (15.4%) as controls (mean [SD] age, 144.3 [7.7] months; 513 [50.7%] males), using the parent interviews from the K-SADS. A total of 3414 individuals (52.0%) were included for the sleep problems analysis, 68 (1.0%) as diagnoses (mean [SD] age, 143.5 [7.5] months; 38 [55.9%] males) and 3346 (50.9%) as controls (mean [SD] age, 143.5 [7.7] months; 1725 [51.6%] males), using the child interviews from the K-SADS.

### Baseline Characteristics of the Training Data Set

The training data set consisted of 12 348 entries for ADHD analysis (818 [6.62%] with ADHD and 11 530 [93.38%] controls) and 39 160 for sleep problems analysis (722 [1.84%] with sleep problems and 38 438 [98.16%] controls). The training data set consisted of 79 individuals with ADHD, of whom 55 (69.6%) were male and 24 (30.4%) were female, and 68 individuals with sleep problems, of whom 38 (56.0%) were male and 30 (44.0%) were female (Table 1; eTable 1 in Supplement 1). When comparing the ADHD diagnoses with the controls, reported as mean (SD), the 30-second sleep records showed that, in the ADHD cohort, the duration of in-bed sleep was 3.9 (0.9) minutes shorter (*P* = .007) and the duration of light sleep was 3.3 (0.8) minutes shorter (*P* = .001). In addition, the duration of being asleep was 8.5 (3.4) minutes shorter (*P* = .001) in the 60-second sleep records; the heart rate records showed that the bedtime heart rate was 1.6 (0.5) bpm higher (*P* < .001) and daytime heart rate was 1.5 (0.7) bpm higher (*P* < .001). Moreover, the maximum heart rate was 1.7 (0.9) bpm higher (*P* < .001), mean heart rate was 1.6 (0.5) bpm higher (*P* < .001), and midline estimating statistics of rhythm heart rate was 1.7 (0.5) bpm higher (*P* < .001). In addition, the sum of calories in the ADHD cohort was 69.4 (28.1) kcal higher (*P* < .001) in the calorie records. The steps records showed that the duration of light activity was 6.5 (0.4) minutes shorter (*P* < .001) in the ADHD cohort and the duration of moderate activity was 15.1 (12.4) minutes longer (*P* < .001).

When comparing the participants with sleep problems diagnoses with the controls, the 30-second sleep records showed that the duration of rapid eye movement sleep was 2.2 (0.1) minutes shorter (*P* < .001) and the waking for short periods was 2.0 (0.2) minutes longer (*P* < .001). Furthermore, the rate of rapid eye movement sleep was 0.9% (0.2) shorter (*P* < .001) and the rate of wake sleep was 0.9% (0.0%) higher (*P* < .001). In addition, the duration of restlessness was 2.1 (0.2) minutes longer (*P* < .001) and the quality of sleep was 0.4% (0.1%) lower (*P* < .001) in 60-second sleep records. Regarding the heart rate records, the bedtime maximum in children with sleep disorders was 2.2 (0.3) bpm higher (*P* < .001) than in the controls. The metabolic equivalents–related records showed that the duration of sedentary activity was 21.5 (7.9) minutes longer (*P* < .001) and the duration of vigorous activity was 3.1 (2.6) minutes shorter (*P* < .001). Regarding the steps records, the sum of bedtime was 18.7 (140.4) steps higher (*P* < .001), the sum of daytime was 818.5 (53.7) steps lower (*P* < .001), and the sum of steps was 799.8 (106.6) steps lower (*P* < .001).

### Model Performance

The performance of the ML models for predicting the ADHD and sleep problems was measured from the mean and highest PPV. In the ADHD diagnosis group, the LightGBM model showed the highest average performance: AUC was 0.791 (95% CI, 0.790-0.792), sensitivity was 0.718 (95% CI, 0.715-0.722), specificity was 0.716 (95% CI, 0.715-0.717), PPV was 0.152 (95% CI, 0.152-0.153), and NPV was 0.973 (95% CI, 0.972-0.973). In the sleep problems group, the LightGBM model showed the highest average performance: AUC was 0.735 (95% CI, 0.735-0.736), sensitivity was 0.718 (95% CI, 0.715-0.720), specificity was 0.628 (95% CI, 0.628-0.629), PPV was 0.035 (95% CI, 0.035-0.036), and NPV was 0.992 (95% CI, 0.992-0.993). The detailed average performance of the ML models is given in Table 2.

**Table 2.** Average Performance Evaluations of Machine Learning Models^a^

Diagnosis	Model	Average performance, mean (SD) [95% CI]				
		AUC	Sensitivity	Specificity	PPV	NPV
ADHD	RF	0.739 (0.011) [0.736-0.742]	0.730 (0.023) [0.724-0.736]	0.634 (0.012) [0.630-0.637]	0.124 (0.004) [0.123-0.125]	0.971 (0.002) [0.970-0.971]
	XGB	0.789 (0.004) [0.788-0.790]	0.719 (0.014) [0.715-0.723]	0.716 (0.005) [0.714-0.717]	0.153 (0.003) [0.152-0.153]	0.973 (0.001) [0.972-0.973]
	LGB	0.791 (0.003) [0.790-0.792]	0.718 (0.014) [0.715-0.722]	0.716 (0.004) [0.715-0.717]	0.152 (0.003) [0.152-0.153]	0.973 (0.001) [0.972-0.973]
Sleep problems	RF	0.641 (0.007) [0.639-0.643]	0.813 (0.018) [0.808-0.818]	0.373 (0.006) [0.372-0.375]	0.024 (0.001) [0.024-0.025]	0.991 (0.001) [0.990-0.991]
	XGB	0.724 (0.003) [0.724-0.725]	0.712 (0.008) [0.710-0.714]	0.614 (0.002) [0.614-0.615]	0.033 (0.000) [0.033-0.034]	0.991 (0.000) [0.991-0.992]
	LGB	0.735 (0.002) [0.735-0.736]	0.718 (0.009) [0.715-0.720]	0.628 (0.002) [0.628-0.629]	0.035 (0.000) [0.035-0.036]	0.992 (0.000) [0.992-0.993]

Abbreviations: ADHD, attention-deficit/hyperactivity disorder; AUC, area under the receiver operating characteristics curve; LGB, light gradient-boosting machine; NPV, negative predictive value; PPV, positive predictive value; RF, random forest; XGB, extreme gradient boosting.

^a^ Each model was trained 50 times per diagnosis; subsequently, all performances of each model were listed and the mean was calculated.

**Figure 2.**
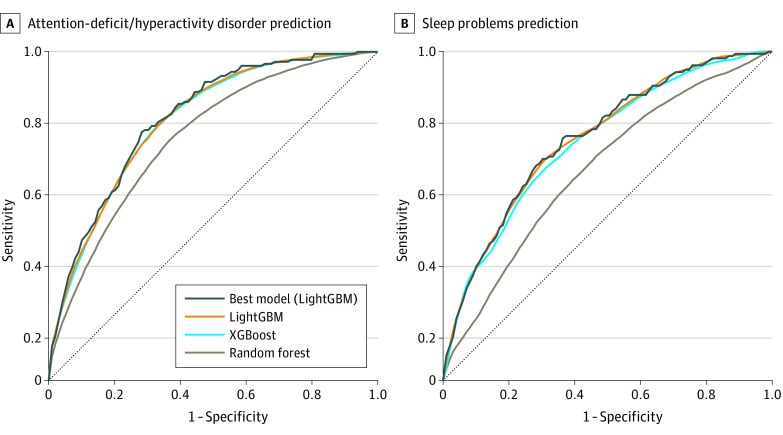
Area Under the Receiver Operating Characteristic (AUC) Curves of Target Diagnoses Prediction Predictions for attention-deficit/hyperactivity disorder (A) and sleep problems (B). The dashed line presents the average performance of the machine learning model. AUC (95% CI) findings for the ADHD population were 0.739 (0.736-0.742) for random forest, 0.789 (0.788-0.790) for extreme gradient boosting (XGBoost), 0.791 (0.790-0.792) for light gradient-boosting machine (LightGBM), and 0.798 for best model (LightGBM). AUC (95% CI) findings for the sleep problems population were 0.704 (0.701-0.707) for random forest, 0.717 (0.714-0.719) for XGBoost, 0.726 (0.723-0.730) for LightGBM, and 0.737 for best model (LightGBM). Each 95% CI was calculated by the list of AUC values from machine learning models.

Figure 2 shows the average predictive performance of the 3 ML models and the highest predictive performance of the ML model. The highest performance was measured from the best LightGBM model for both the ADHD and sleep problems diagnoses. For the ADHD group, the highest performance AUC was 0.798, sensitivity was 0.756, specificity was 0.716, PPV was 0.159, and NPV was 0.976. For the sleep problems group, AUC was 0.737, sensitivity was 0.743, specificity was 0.632, PPV was 0.036, and NPV was 0.992.

### Feature Interpretation

**Figure 3.**
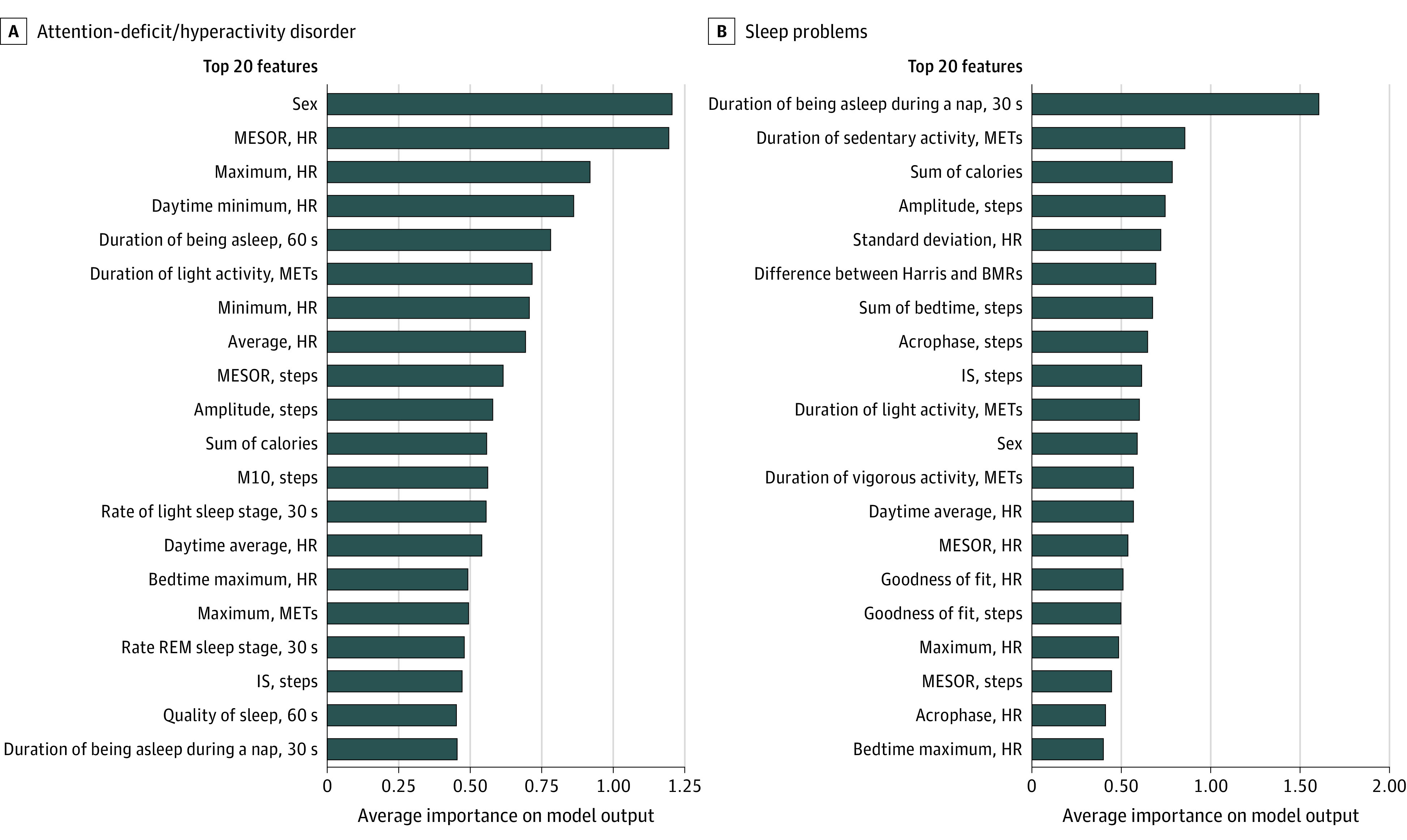
Summary of Shapley Additive Explanations Importance by the Best Prediction Model The lists of feature importance for attention-deficit/hyperactivity disorder (A) and sleep problems (B) are arranged in descending order. The higher value (average importance) indicates that the feature affects the model output (probability) to be higher. BMR indicates basal metabolic rate; Harris, Harris-Benedict equation for calculating BMR; HR, heart rate; IS, interdaily stability; M10, most active 10-hour period; MESOR, midline estimating statistics of rhythm; METs, metabolic equivalents; and REM, rapid eye movement.

The feature importance was measured by the Shapley Additive Explanations values. Figure 3 shows the top 20 important features by the best predictive model for each diagnosis. For the ADHD group, the predictive model included sex (55 [69.6%] males; *P* < .001), midline estimating statistics of rhythm heart rate (mean [SD], 84.4 [8.0] bpm; *P* < .001), maximum heart rate (mean [SD], 103.5 [8.5] bpm; *P* < .001), daytime minimum heart rate (mean [SD], 59.8 [6.4] bpm; *P* = .12), and duration of being asleep 60 seconds (mean [SD], 465.4 [79.8] minutes; *P* = .001). For the sleep problems group, the predictive model included duration of being asleep during a nap, 30 seconds (mean [SD], 47.0 [40.7] minutes; *P* < .001); duration of sedentary activity, metabolic equivalents (mean [SD], 405.9 [103.3] minutes; *P* < .001); sum of calories (mean [SD], 2026.2 [437.5] kcal; *P* = .35); amplitude, steps (mean [SD]; 408.2 [206.2] steps; *P* = .10); and heart rate, SD (mean [SD], 15.3 [3.1] bpm; *P* = .70). The detailed average importance of the features is reported in eTable 2 in Supplement 1.

## Discussion

This study aimed to predict ADHD and sleep problems in children based on wearable device data, anthropometrics, sex, and K-SADS data from the ABCD study. To our knowledge, this study is the first attempt to construct an ML model to predict the diagnosis of specific psychiatric disorders using wearable data from the ABCD study. In previous studies, the prevalence of various sleep-related problems increased in patients with ADHD^31,32,33^; children with ADHD showed longer light sleep and had poorer quality of sleep than those in the control cohorts.^34^ In this study, the patients with ADHD showed shorter sleep times and higher intensities of daytime activity compared with the controls. The problems of attention, arousal, and sleep control of patients with ADHD share some common features regarding neuroanatomical functions.^33,34,35^

Children report various types of sleep problems,^36^ and all participants classified as having sleep problems in the ABCD study reported insomnia. However, children with sleep problems napped longer, were more sedentary, and engaged in less vigorous activity than those in the control group. This finding suggests that the sleep problem diagnoses show digital phenotypes that contradict circadian and homeostatic physiologic processes.^37^

In this study, it was possible to confirm the diagnostic potential by using the processed data to ML training by applying circadian analysis to various data acquired with a wearable device. In addition, the ML analysis method enabled us to identify features that could predict mental disorders.^38,39,40,41^ We found that many of the top-ranked features for ADHD prediction were variables related to heart rate. Heart rate is one of the indicators that can estimate autonomic nervous system activity^42,43^; an increase in mean heart rate reflects the relative dominance of the sympathetic nervous system.^44^ The highest-ranked features for predicting sleep problems were variables related to nap and activity patterns. The difference in feature importance patterns between the ADHD and sleep problems groups helps to understand sleep problems based on the digital phenotypes and provides hints for practical therapeutic intervention.

Considering the general use of wearable devices, missing values are inevitable. Compared with other models, the extreme gradient boosting and LightGBM models are more useful in predicting diagnoses because they can reflect such missing values. However, we should consider that the precision is relatively low compared with other prediction performances, and there are many false-positives. These results suggest that there may be limitations in advancing psychiatric diagnosis performance centering on wearable data. Wearable devices, like passive digital phenotypes, have the advantage of being able to automatically and invisibly secure data in children’s daily lives. Therefore, the results of this study are most useful for the early detection of high-risk groups or disease screening in daily life rather than the application to diagnose ADHD or sleep problems in the medical field.

In previous ABCD studies, predictions using survey data were found to have relatively high accuracy with an AUC of 0.80 or higher.^22,45,46,47^ However, while questionnaires directly evaluate the characteristics of diseases and require intention and effort, wearable data can be a useful digital health technology because they can provide information related to life patterns or physiologic characteristics in daily life.^48^ In particular, because wearable data derive clinical value by processing data based on circadian rhythm, they are more meaningful.^49,50^

### Limitations

This study has limitations. First, there was a highly imbalanced class problem due to insufficient prediction classes. Therefore, the model should be strengthened by securing additional cohorts. Second, wearable device data do not guarantee accurate measurement values. Therefore, the wearable data may contain errors before processing.^51^ For example, wearable devices tend to be underestimated in a controlled environment and overestimated in a free environment.^52^ Third, the diagnosis of mental disorders should be multifaceted based on expert knowledge and clinical experience. Therefore, the ML model based on the diagnosis using the K-SADS may be limited.^53^ Fourth, wearable devices can measure some sleep-related variables directly; however, overestimation must be considered when interpreting ML performance results. Nevertheless, the circadian characteristic feature data were useful for predicting ADHD and sleep problems in children.^41,54,55,56,57^

## Conclusions

In this diagnostic study of ADHD and sleep problems in children, we were able to develop a predictive ML model that appears to be applicable for early detection or screening using digital phenotypes. Generating and processing circadian rhythm–based features and using ML models for predicting ADHD and sleep problems is beneficial for early intervention and prevention in daily life. The accumulation of clinical and digital phenotyping data will enable future scholars to make advanced prediction models and develop useful criteria for the early assessment and intervention of psychiatric disorders in children.

## Notes

Supplement 1. **eMethods 1.** Study Population Inclusion and Exclusion Criteria **eMethods 2.** Process of Making Wearable Dataset **eMethods 3.** Process of Making Training Dataset **eTable 1.** Basic Characteristics and Demographics of the ABCD Study Participants **eTable 2.** Top 10 Shapley Additive Explanations (SHAP) Importance Summary **eAppendix.** Equations

Supplement 2.
Data Sharing Statement
