# Supplementary material for: Machine Learning–Based Prediction of Attention-Deficit/Hyperactivity Disorder and Sleep Problems With Wearable Data in Children
Source: JAMA Netw Open. 2023 Mar 17;6(3):e233502. doi: 10.1001/jamanetworkopen.2023.3502 (PMC10024208; doi:10.1001/jamanetworkopen.2023.3502)
Supplement: Supplement 1. — eMethods 1. Study Population Inclusion and Exclusion Criteria eMethods 2. Process of Making Wearable Dataset eMethods 3. Process of Making Training Dataset eTable 1. Basic Characteristics and Demographics of the ABCD Study Participants eTable 2. Top 10 Shapley Additive Explanations (SHAP) Importance Summary eAppendix. Equations [file jamanetwopen-e233502-s001.pdf]

Supplementary Online Content

Kim WP, Kim HJ, Pack SP, Lim JH, Cho CH, Lee HJ. Machine learning–based prediction of attention-deficit/hyperactivity disorder and sleep problems with wearable data in children. *JAMA Netw Open*. 2023;6(3):e233502. doi:10.1001/jamanetworkopen.2023.3502

eMethods 1. Study Population Inclusion and Exclusion Criteria

eMethods 2. Process of Making Wearable Dataset

eMethods 3. Process of Making Training Dataset

eTable 1. Basic Characteristics and Demographics of the ABCD Study Participants

eTable 2. Top 10 Shapley Additive Explanations (SHAP) Importance Summary

eAppendix. Equations

This supplementary material has been provided by the authors to give readers additional information about their work.

© 2023 Kim WP et al. *JAMA Network Open.*

eMethods 1. Study Population Inclusion and Exclusion Criteria

Study Population Inclusion and Exclusion Criteria

The attention deficit hyperactivity disorder (ADHD) and sleep problems inclusion was extracted from the diagnostic group from the Kiddie Schedule for Affective Disorders and Schizophrenia Present and Lifetime Version for DSM-5 (K-SADS). The unique identifier ("subjectkey" in the Adolescent Brain Cognitive Development [ABCD] study) of those without any diagnoses (comorbidities) and symptoms were extracted and set as controls that accordingly did not contain any diagnoses (comorbidities) and symptoms, while diagnoses were set to include all of the various symptoms.

For the ADHD analysis, the diagnoses were extracted using the unique identifier marked with "1" as the diagnostic results of 6,571 individuals from the parent interviewee of the K-SADS. Continuously, the extracted controls were marked "0" as the diagnostic results. First, 2,603 individuals with symptoms were excluded from the controls. Second, 2,025 individuals with other diagnoses were excluded from the final study inclusion. Third, 355 individuals with comorbidities were excluded from the diagnoses. Fourth, 498 individuals who had missing wearable data were excluded from both the diagnoses and controls. Finally, for the ADHD analysis, 1,090 individuals (79 as diagnoses; 1,011 as controls) were included in the final study inclusion.

In the same way, for the sleep problems analysis, the diagnoses were extracted using the unique identifier marked with "1" as the diagnostic results of 6,571 individuals from the child interviewee of the K-SADS. Continuously, the controls were extracted with marked "0" as the diagnostic results. First, 1,062 individuals with symptoms were excluded from the controls. Second, 1,482 individuals with other diagnoses were excluded from the final study inclusion. Third, 140 individuals with comorbidities were excluded from the sleep problems analysis. Fourth, 473 individuals who had missing wearable data were excluded from both the diagnoses and controls. Finally, for the sleep problems analysis, 3,414 individuals (68 as diagnoses; 3,346 as controls) were included in the final study inclusion.

© 2023 Kim WP et al. *JAMA Network Open.*

eMethods 2. Process of Making Wearable Dataset

Process of Making Wearable Dataset from The Nine Basic Features

With wearable devices, the nine basic feature data are largely divided into heart rate recorded in 30-second to one-minute increments, four stages of sleep for the 30-second records, four stages of sleep for the 60-second records, and physical activity data. There were up to seven mobile data files per cohort.

The maximum, minimum, and average heart rates were calculated, and the 30-second sleep was classified into four stages (light, deep, random eye movement [REM], and wake) and the 60-second sleep into three stages (asleep, restless, and awake), which were expressed in one-hot encoding. This process aims to derive a summary of what stages of sleep the user was in per hour. It also indicates the duration of each sleep phase per hour.

For circadian rhythm analysis, this study set a sliding window at three-day intervals to reflect the circadian rhythm in wearable device data and added estimated values of midline estimating statistics of rhythm (MESOR), amplitude, and acrophase to the circadian feature data through the Cosinor analysis.

For the objective analysis of circadian physical activity, intradaily variability, interdaily stability, L5, M10, and relative amplitude[19] were calculated using the number of recorded steps. The detailed equations are shown in **eEquations 4–6**.

Using the physical information of each cohort, the basal metabolic rate and the difference with the daily calorie consumption were calculated. The basal metabolic rate was calculated using the equations by Mifflin-St Jeor,[21] Katch-McArdle,[22] and Harris-Benedict.[23] The detailed equations are shown in **eEquations 1–3**.

From the sleep data, the daily duration and rate of each sleep phase and total sleep quality were calculated based on four sleep stages recorded for 30 seconds and three sleep phases recorded for 60 seconds. Furthermore, this study set bedtime (9 PM to 5 AM) and daytime (6 AM to 8 PM) to identify any naps occurring in the sleep of the 60-second records. Finally, the number of steps recorded at bedtime and daytime was summed, and the minimum, maximum, and average heart rate were calculated to be added to the circadian feature data.

© 2023 Kim WP et al. *JAMA Network Open.*

eMethods 3. Process of Making Training Dataset

Process of Making Training Dataset

The heart rate, sleep, and activity data corresponding to each date were joined with a unique identifier ("subjectkey" in the ABCD study). The missing values in the nap data were substituted with 0. The substituted value indicated that there was no nap on the day. Furthermore, the nine basic wearable data were expanded to 64 circadian characteristic data through circadian rhythm analysis with a sliding window at three-day intervals.

In the Parent and Child K-SADS diagnostic results of 6,571 patients were used. For the ADHD analysis, 5,481 individuals were excluded. The final inclusion was 1,090 individuals (79 as diagnoses; 1,011 as controls). In the same way, for the sleep problems analysis, a total of 3,157 individuals were excluded. The final inclusion was 3,414 individuals (68 as diagnoses; 3,346 as controls).

The training data were extracted from a wearable data comprising 64 circadian characteristics using the unique identifier of the subject to be analyzed. During the inclusion process, matched 21 days of wearable data in each group by "subjectkey" were linked. Finally, the training data for the ADHD consisted of 12,348 wearable data (1,090 individuals; 79 as diagnoses, 1,011 as controls), and the sleep problems consisted of 39,160 wearable data (3,414 individuals; 68 as diagnoses, 3,346 as controls).

When training each model based on the wearable data generated in **eMethod 3**, 20% of the training data was extracted as the hold-out test set. In the remaining 80% of the data, 70% was used for the training set, and 10% was used for the validation set.

© 2023 Kim WP et al. *JAMA Network Open.*

eTable 1. Basic Characteristics and Demographics of the ABCD Study Participants

Characteristic	ABCD Study, Release 3.0, November. 2, 2020				
		By Parent K-SADS (Diagnosis)		By Child K-SADS (Diagnosis)	
	Total (n = 6571)	Control (n = 1011)	ADHD (n = 79)	Control (n = 3346)	Sleep Problems (n = 68)
Age, mean (SD)					
Year	12.0 (0.6)	12.0 (0.6)	12.0 (0.7)	12.0 (0.6)	12.0 (0.6)
Month	143.5 (7.7)	144.3 (7.7)	144.5 (8.1)	143.5 (7.7)	143.5 (7.5)
Sex; count (%)					
Male	3467 (52.8)	513 (50.7)	55 (69.6)	1725 (51.6)	38 (55.9)
Female	3104 (47.2)	498 (49.3)	24 (30.4)	1621 (48.4)	30 (44.1)
Language, count (%)					
English	5967 (90.8)	949 (93.9)	76 (96.2)	3149 (94.1)	63 (92.6)
Spanish	456 (6.9)	44 (4.4)	2 (2.5)	126 (3.8)	4 (5.9)
Mandarin	17 (0.3)	4 (0.4)	0 (0.0)	12 (0.4)	0 (0.0)
Russian	12 (0.2)	2 (0.2)	1 (1.3)	4 (0.1)	0 (0.0)
Others (refused to answer, did not answer)	119 (1.8)	12 (1.2)	0 (0.0)	55 (1.6)	1 (1.5)
Race (parent), count (%)					
White	4838 (73.6)	803 (79.4)	62 (78.5)	2704 (80.8)	62 (91.2)
Black or African American	769 (11.7)	98 (9.7)	6 (7.6)	287 (8.6)	3 (4.4)
Asian	197 (3.0)	30 (3.0)	2 (2.5)	84 (2.5)	0 (0.0)
Others	551 (8.4)	33 (3.3)	8 (10.1)	220 (6.6)	2 (2.9)
Multiple	45 (0.7)	39 (3.9)	0 (0.0)	21 (0.6)	0 (0.0)
Unsure	171 (2.6)	8 (0.8)	1 (1.3)	30 (0.9)	1 (1.5)
Marital status, count (%)					
Married	4518 (68.8)	778 (77.0)	59 (74.7)	2536 (75.8)	49 (72.1)
Divorced	666 (10.1)	91 (9.0)	5 (6.3)	314 (9.4)	6 (8.8)
Never married	605 (9.2)	65 (6.4)	5 (6.3)	224 (6.7)	5 (7.4)
Living with partner	401 (6.1)	55 (5.4)	4 (5.1)	147 (4.4)	4 (5.9)

© 2023 Kim WP et al. *JAMA Network Open.*

Separated	245 (3.7)	16 (1.6)	4 (5.1)	87 (2.6)	3 (4.4)
Widowed	72 (1.1)	3 (0.3)	2 (2.5)	27 (0.8)	1 (1.5)
Others (refused to answer, did not answer)	64 (1.0)	3 (0.3)	0 (0.0)	11 (0.3)	0 (0.0)
Working status, count (%)					
Working now (full+part time)	4997 (76.0)	834 (82.5)	67 (84.8)	2644 (79.0)	51 (75.0)
Stay at home	889 (13.5)	124 (12.3)	6 (7.6)	461 (13.8)	10 (14.7)
Looking for work	200 (3.0)	15 (1.5)	2 (2.5)	77 (2.3)	0 (0.0)
Disabled (permanently or temporarily)	127 (1.9)	9 (0.9)	1 (1.3)	45 (1.3)	2 (2.9)
Student	78 (1.2)	5 (0.5)	1 (1.3)	27 (0.8)	1 (1.5)
Others (retired, sick leave, refused to answer)	280 (4.3)	24 (2.4)	2 (2.5)	92 (2.8)	4 (0.5)
Total house income (past 12 months, before taxes), count (%)					
~$24999	649 (9.9)	56 (5.5)	4 (5.1)	208 (6.2)	2 (2.9)
$25000~$50000	750 (11.4)	90 (8.9)	9 (11.4)	294 (8.8)	12 (17.6)
$50000~$100000	1696 (25.8)	267 (26.4)	22 (27.8)	898 (26.8)	22 (32.4)
$100000~$199999	2089 (31.8)	349 (34.5)	30 (38.0)	1235 (36.9)	20 (29.4)
$200000~	887 (13.5)	109 (18.7)	11 (13.9)	531 (15.9)	8 (11.8)
Others (refuse to answer, did not answer)	500 (7.6)	60 (5.9)	3 (3.8)	180 (5.4)	4 (5.9)
Another household with child, count (%)					
No	5788 (88.1)	931 (92.1)	70 (88.6)	3004 (89.8)	59 (86.8)
Yes	734 (11.2)	76 (7.5)	9 (11.4)	334 (10.0)	9 (13.2)
Others (refused to answer, did not answer)	49 (0.8)	4 (0.4)	0 (0.0)	8 (0.2)	0 (0.0)
Best household (If “yes” above)					
Other biological parents	527 (71.8)	58 (76.3)	9 (69.2)	247 (74.0)	6 (66.7)
Grandmother	132 (18.0)	13 (17.1)	4 (30.8)	65 (19.5)	1 (11.1)
Aunt	24 (3.3)	2 (2.6)	0 (0.0)	7 (2.1)	1 (11.1)
Others (step parent, uncle, refused to answer)	51 (6.9)	3 (3.9)	0 (0.0)	15 (4.4)	1 (11.1)

© 2023 Kim WP et al. *JAMA Network Open.*

People in the house (past 2 months)					
0	27 (0.4)	3 (0.5)	0 (0.0)	14 (0.4)	0 (0.0)
1~3	1105 (18.8)	124 (12.3)	15 (19.0)	488 (14.6)	15 (22.1)
4~6	4708 (71.6)	772 (76.4)	59 (74.7)	2519 (75.3)	44 (64.7)
7~9	552 (8.4)	93 (9.2)	5 (6.3)	278 (8.3)	9 (13.2)
10~	53 (0.8)	8 (0.8)	0 (0.0)	23 (0.7)	0 (0.0)
Others (refused to answer, did not answer)	126 (1.9)	11 (1.1)	0 (0.0)	24 (0.7)	0 (0.0)

Abbreviations: ABCD, Adolescent Brain Cognitive Development; ADHD, attention deficit hyperactivity disorder; K-SADS, Kiddie Schedule for Affective Disorders and Schizophrenia Present and Lifetime Version for DSM-5; SD, standard deviation

© 2023 Kim WP et al. *JAMA Network Open.*

eTable 2. Top 10 Shapley Additive Explanations (SHAP) Importance Summary

Diagnosis	Model	Feature Names	Average Importance (mean [SD] 95% CI)
ADHD	RF	Sex	0.01716 [0.00153] (0.01674–0.01759)
		Duration of light activity, METs	0.01680 [0.00147] (0.01639–0.01721)
		MESOR, HR	0.01278 [0.00202] (0.01222–0.01334)
		Sum of calories	0.00902 [0.00186] (0.00850–0.00953)
		Minimum, HR	0.00898 [0.00215] (0.00838–0.00957)
		Maximum, HR	0.00747 [0.00177] (0.00698–0.00796)
		Bedtime average, HR	0.00738 [0.00167] (0.00692–0.00784)
		Daytime average, HR	0.00689 [0.00199] (0.00634–0.00744)
		MESOR, steps	0.00662 [0.00170] (0.00615–0.00710)
		Average, HR	0.00629 [0.00158] (0.00585–0.00673)
	XGB	Sex	0.12405 [0.00424] (0.12288–0.12523)
		Duration of light activity, METs	0.08953 [0.00386] (0.08846–0.09060)
		MESOR, HR	0.08403 [0.00876] (0.08160–0.08646)
		Sum of calories	0.05979 [0.00567] (0.05821–0.06136)
		Minimum, HR	0.04886 [0.00778] (0.04671–0.05102)
		Bedtime average, HR	0.04397 [0.00480] (0.04264–0.04530)
		Daytime average, HR	0.04376 [0.00527] (0.04230–0.04522)
		IS, steps	0.04373 [0.00346] (0.04277–0.04469)
		Quality of sleep, 60-seconds	0.04089 [0.00280] (0.04011–0.04166)
		Maximum, METs	0.03994 [0.00429] (0.03875–0.04113)
	LGB	MESOR, HR	0.14641 [0.00836] (0.14409–0.14873)
		Sex	0.13380 [0.00330] (0.13288–0.13471)
		Duration of light activity, METs	0.10430 [0.00378] (0.10325–0.10535)
		Sum of calories	0.08103 [0.00657] (0.07921–0.08285)
		Maximum, HR	0.07964 [0.00446] (0.07840–0.08087)
		Bedtime average, HR	0.06424 [0.00554] (0.06271–0.06578)
		IS, steps	0.06273 [0.00305] (0.06188–0.06357)
		Rate of REM sleep, 30-seconds	0.05828 [0.00281] (0.05750–0.05906)
		Quality of sleep, 60-seconds	0.04979 [0.00200] (0.04923–0.05034)
		Duration of sedentary activity, METs	0.04649 [0.00283] (0.04571–0.04728)
Sleep problems	RF	Duration of being asleep, 30 seconds	0.02780 [0.01105] (0.02473–0.03086)
		Duration of sedentary activity, METs	0.02336 [0.00841] (0.02103–0.02569)
		MESOR, steps	0.02286 [0.01012] (0.02006–0.02567)
		RA, steps	0.01641 [0.00938] (0.01381–0.01901)
		Sum of bedtime, steps	0.01490 [0.00729] (0.01288–0.01692)
		M10, steps	0.01290 [0.00800] (0.01068–0.01512)
		Sum of steps	0.01082 [0.00765] (0.00870–0.01294)
		Quality of sleep, 60-seconds	0.00976 [0.00459] (0.00849–0.01103)

© 2023 Kim WP et al. *JAMA Network Open.*

		Goodness of fit, HR	0.00957 [0.00375] (0.00853–0.01061)
		Goodness of fit, steps	0.00888 [0.00344] (0.00793–0.00983)
	XGB	Duration of being asleep during a nap, 30-seconds	0.55752 [0.15608] (0.51425–0.60078)
		Standard deviation, HR	0.49666 [0.13982] (0.45790–0.53541)
		Duration of sedentary activity, METs	0.46152 [0.12445] (0.42702–0.49602)
		Sum of bedtime, steps	0.35449 [0.11280] (0.32323–0.38576)
		Sum of calories	0.34547 [0.09878] (0.31809–0.37285)
		Goodness of fit, HR	0.34494 [0.08847] (0.32041–0.36946)
		MESOR, steps	0.34418 [0.10646] (0.31467–0.37369)
		Goodness of fit, steps	0.32816 [0.09375] (0.30218–0.35415)
		Sex	0.32356 [0.10635] (0.29409–0.35304)
		Duration of vigorous activity, METs	0.31856 [0.11226] (0.28744–0.34967)
	LGB	Duration of asleep during a nap, 30 seconds	1.04284 [0.28856] (0.96286–1.12282)
		Duration of sedentary activity, METs	0.88615 [0.23023] (0.82234–0.94997)
		Standard deviation, HR	0.87000 [0.22508] (0.80761–0.93239)
		Sum of calories	0.77256 [0.22361] (0.71058–0.83454)
		Duration of bedtime, steps	0.75570 [0.19824] (0.70075–0.81065)
		Goodness of fit, steps	0.72187 [0.19963] (0.66653–0.77720)
		MESOR, steps	0.63304 [0.20841] (0.57527–0.69080)
		Goodness of fit, HR	0.62416 [0.12011] (0.59086–0.65745)
		Duration of vigorous activity, METs	0.59149 [0.19341] (0.53788–0.64510)
		Acrophase, HR	0.55291 [0.15492] (0.50997–0.59585)

*The mean, standard deviation, and 95% confidence interval of the feature importance were calculated from all trained machine learning models for each diagnosis.

**All scores were accumulated and sorted in the same order by the model (RF, XGB, LGB). Finally, we partially selected the top 10 features to summarize SHAP importance scores.

Abbreviations: CI, confidence interval; ADHD, attention deficit hyperactivity disorder; RF, random forest; XGB, extreme gradient boosting; LGB, light gradient-boosting machine; HR, heart rate; REM, rapid eye movement; MESOR, midline estimating statistics of rhythm; METs, metabolic equivalents; IS, interdaily stability; IV, intradaily variability; M10, most active ten-hour period; RA, relative activity.

© 2023 Kim WP et al. *JAMA Network Open.*

eAppendix. Equations

eEquation 1. Formula of Basal Metabolic Rate by Miffiln–St Jeor

$$\text{BMR} = 10 \times \text{weight(kg)} + 6.25 \times \text{height(cm)} - 5 \times \text{age (y)} + S$$

$$S = \begin{cases} +5, & male \\ -161, & female \end{cases}$$

eEquation 2. Formula of Basal Metabolic Rate by Katch–McArdle

$$\text{BMR} = 370 + (21.6 \times \text{LBM})$$

$$\text{Lean body mass (LBM)} = \begin{cases} 0.407 \times \text{weight (kg)} + 0.267 \times \text{height (cm)} - 19.2, & male \\ 0.252 \times \text{weight (kg)} + 0.473 \times \text{height (cm)} - 48.3, & female \end{cases}$$

eEquation 3. Formula of Basal Metabolic Rate by Harris–Benedict

$$BMR = \begin{cases} 88.362 + (13.397 \times weight\ [kg]) + (4.799 \times height\ [cm]) - (5.677 \times age\ [y]), & male \\ 447.593 + (9.247 \times weight\ [kg]) + (3.098 \times height\ [cm]) - (4.330 \times age\ [y]), & female \end{cases}$$

eEquation 4. Formula of Interdaily Stability (IS)

$$IS = \frac{n \sum_{h=1}^{p} (\overline{x_h} - \overline{x})^2}{p \sum_{i=1}^{n} (x_i - \overline{x})^2}$$

n: total number of data

p: number of data per day

$\overline{x_h}$: hourly means

$\overline{x}$: mean of all data

eEquation 5. Formula of Intradaily Variability (IV)

$$IV = \frac{n \sum_{i=2}^{n} (x_i - x_{i-1})^2}{(n-1) \sum_{i=1}^{n} (x_i - \overline{x})^2}$$

n: total number of data

eEquation 6. Formula of Relative Amplitude (RA)

$$RA = \frac{M10 - L5}{M10 + L5}$$

© 2023 Kim WP et al. *JAMA Network Open.*

$L5$: least active five-hour period

$M10$: most active 10-hour period

© 2023 Kim WP et al. *JAMA Network Open.*
