# Supplementary material for: Machine Learning–Based Prediction of Attention-Deficit/Hyperactivity Disorder and Sleep Problems With Wearable Data in Children
Source: JAMA Netw Open. 2023 Mar 17;6(3):e233502. doi: 10.1001/jamanetworkopen.2023.3502 (PMC10024208; doi:10.1001/jamanetworkopen.2023.3502)
Supplement: Supplement 2. — Data Sharing Statement [file jamanetwopen-e233502-s002.pdf]

Data Sharing Statement

Kim. Machine Learning-Based Prediction of Attention-Deficit/Hyperactivity Disorder and Sleep Problems With Wearable Data in Children. *JAMA Netw Open*. Published March 17, 2023. doi:10.1001/jamanetworkopen.2023.3502

Data
Data available: No

Additional Information
Explanation for why data not available: We are unable to share the data directly since an agreement with the NIH is required to access the data (see https://nda.nih.gov/abcd/). We will be able to share the code used for the analysis if a request for code alone is made.
